# The Professional Code of Ethics and Some of Its Privileges

**Published:** 1910-01-15

**Authors:** George W. Clapp

**Affiliations:** New York


### THE PROFESSIONAL CODE OF ETHICS AND SOME OF ITS PRIVILEGES.

BY GEORGE W. CLAPP, D.D.S., NEW YORK.

No discussion of ethics can be intelligent which is not founded on agreed upon definition. The one definition which all the world has so far sanctioned is generally known as The Golden Rule. It reads, "Whatsoever ye would that men should do unto you, do ye even so to them." Let us then start with this Golden Rule as an agreed on definition of ethics, and investigate how far it concerns itself with the conduct of dental practice, whether it restricts us from profitable activities, and whether we need to overlap the few real barriers it erects.

### THE NEW CODE OF ETHICS OF THE CHICAGO ODONTOGRAPHIC SOCIETY.

Section I. In his dealings with patients and with the profession, the conduct of the dentist should be in accordance with The Golden Rule, both in its letter and its spirit.

Section II. It is unprofessional for a dentist to advertise by hand-bills, posters, circulars, cards, signs, or in newspapers or other publications, calling attention to special methods of practice, or claiming excellence over other practitioners, or to use display advertisements of any kind. This does not exclude a practitioner from using professional cards of suitable size with the name, titles, address and telephone number, printed in modest type, nor having the same character of card in a newspaper. Neither does it prevent a practitioner who confines himself to a specialty from merely announcing his specialty on his professional card.

Section III. It is unprofessional for dentists to pay or accept commissions on fees for professional services, or on prescriptions or other articles supplied to patients by pharmacists or others.

Section IV. One dentist should not disparage the services of another to a patient. Criticism of work which is apparently defective may be unjust through the lack of knowledge of the conditions under which the work was performed. The duty of the dentist is to remedy any defect without comment.

Section V. If a dentist is consulted in an emergency by the patient of another practitioner who is temporarily absent from his office, the duty of the dentist so consulted is to relieve the patient of any immediate disability by temporary service only, and then refer the patient back to the regular dentist.

Section VI. When a dentist is called in consultation by a fellow practitioner, he should hold the discussions in the consultation as confidential and under no circumstances should he accept charge of the case without the request of the dentist who has been attending it.

Section VII. The dentist should be morally, mentally and physically clean, and honest in all his dealings with his fellow man, as comports with the dignity of a cultured and professional gentleman.

This Code of Ethics is a moral code. It sets bounds to our activities in certain lines and says "beyond these limits you should not go; but up to this point you may conduct your practice as seems to you best. There is ample room for all your activities within these limits, and you will advance your own interests more truly by not going outside them." Let us see whether this is true.

This Code concerns itself with these activities in practice which affect the rights of others; with the rights of patients on the one hand and the rights of fellow practitioners on the other. We are supposed to deal in honor with our patients, "as we would that they should do unto us," and are enjoined from taking undue advantage of any fellow practitioner.

All Codes say nothing about the dentist's duties to himself. He is supposed to recognize these and to be able to discharge them intelligently without such assistance. Yet a vast number of dentists have not recognized them. They seem to think that because the duties toward self are not specified in the Code, they do not exist, and they seem to have gone through life blind to these things simply because they were not specifically laid out in print before them. *Much of the criticism of the Code comes from dentists who have assumed that the Code was all of dentistry and have not exercised under it that freedom which it grants.*

The Code leaves open to all dentists the very best of practice building methods and the best of methods for handling the business side of practice. Not only this, but sections one, four, five, six and seven safeguard him from straying into practices which are unwise, from a business building standpoint, and thereby actually benefit him. In the fields of commercial business, courtesy and square dealing are assets of no small importance.

What are these practice-building methods which are left open to us by the Code? How large a part would these methods play in successful practice building if there were no Code and we were free to follow, unrestricted, our own suggestions? Could we build desirable practices without these methods?

There will always be differences of opinion as to what constitutes a desirable practice, but for the sake of clearness some standard must be taken. The writer suggests the following restrictions before a practice can be called desirable:

1. It must be conducted on honor.

2. It must be financially profitable, yielding its costs, a good living and a satisfactory sum of annual saving.

3. It must serve the class of people with whom the dentist likes to associate and exhibit the quality of work he is happy in doing.

It is not too much to say that the activities essential to the conduct of a desirable practice, as described here, are permitted by the Code, and that even were there no Code and we were free to wander unrestricted about the fields of dental endeavor, he would exhibit the keenest business sense who wove into the fibre of his practice those very activities. Let us look at their business values.

The dentist is permitted to make his own personal appearence as attractive and pleasing as he sees fit. Let no one underestimate the importance of this. It may seem so small and ordinary a matter as to be hardly worth mentioning, but many a dentist who regards it as unimportant would profit materially by giving it the attention it deserves. Clean clothes, clean linen and cleanliness of person have won many a desirable patient. And the lack of them has lost to the careless dentist just those people whose accounts would be most worth having. A white operating coat is inexpensive, but if carefully kept it makes a favorable impression, out of all proportion to its cost.

Two men recently went into the New York office of the Regal Motor Car Co., to buy an automobile. They thought favorably of the Regal, but went out without purchasing *because the sales manager wore a celluloid collar.* Said the man with the money, "That didn't look very prosperous to me, I'd rather give someone else my money." And he did. A celluloid collar cost that salesman a $1,250 sale.

Not far from the office where this is written practises a dentist of unusual professional skill, who seeks to serve the best class of patients. No criticism of his professional services has ever reached the writer's ears, but some unfortunate personal habits arouse unfavorable comment. He is so addicted to the use of tobacco that his person carries the odor constantly; he is careless about washing his hands when they become slightly soiled during any part of the work; and he frequently puts his hands into his pockets and then operates without washing them. So keen are patients becoming that these habits are unfavorably commented on even by his friends, who know nothing of dentistry save from the patient's side. An attractive personal appearance is permissible to every dentist, under the Code.

Second. The dentist may have an attractive office.

However humble may be its furnishings it can be kept clean and neat. This apparently small item has more influence on the building of a practice than might at first be thought. One of the most financially successful dentists in this land attributes no small part of his practice to the fact that his office was kept immaculate. In a town of only 1,200 people it paid him well to keep an extra helper whose sole duty was to keep his offices clean.

Inquiries among patients show that the value of a neat office as a practice getter is very great. They are displeased by an untidy office and they form an unfavorable opinion of the dentist who permits it. The writer knows of cases where people who would have been most desirable patients have gone away from an office without making an appointment, simply because an untidy waiting-room seemed to them an indication of an unclean dentist.

An immaculate office, which keen business judgment would indicate as warranted in a practice, is permitted by the Code.

Third. The dentist's manner may be as winning as he can make it. There are far more practice-building possibilities in a proper manner than is suspected by many dentists. They are so accustomed to their side of the operation that they forget the patient's frame of mind. Sometimes they have occasion to recall it. A prominent dentist recently said in a dental meeting, "I haven't appreciated the value of a sympathetic manner. Recently I had occasion to have one of my own teeth filled. My son did the work, and the flippant manner in which he spoke of it got on my nerves. *I regarded it much more seriously than he did.*

The patient generally approaches any dental operation with considerable trepidation. Much of the fear is mental and most of it can be allayed by the dentist's manner and words. When the mental terror is allayed, the patient usually has no difficulty in bearing the real pain from any ordinary operation.

The dentist who so keeps in mind the patient's side of the operation that his manner will be sympathetic and reassuring has one of the greatest assets in practice building. The writer has had work done by such men and by equally skillful men who showed no sympathy. So great is the difference in similar operations that he would go far to be sure of the visible, kindly consideration.

A tactful manner, then, is an asset comparable to courtesy which the leading business organizations regard as of first importance, and which we are free to develop to the best of our ability and to exercise in the daily tasks of practice. The New York Central Railroad recently issued to its thousands of employees a circular laying great stress on the business value of courtesy on the part of every employee, no matter how humble. This was one of the first official acts of the present President.

Fourth. The dentist may perfect himself in painless methods. It is very easy for us to be stoical and minimize the pain as long as someone else must bear any pain which befalls. But when a dentist sits down to have one of his own teeth filled, there suddenly comes to his mind a keen perception of what the average patient undergoes at his hands.

For nearly all of the dental operations once painful, painless methods are now possible. General and local anesthetics, skilfully administered, rob much of the work of its terrors and also shorten very materially the time required.

Save ignorance, no other one thing keeps so many people away from the dental office as the fear of being hurt. The dentist who does good work without inflicting severe pain usually has no difficulty in securing plenty of work; and if he is a good business man he usually gets remunerative fees. If it could suddenly dawn on the minds of the public that all dental work could be done with the infliction of so little pain that it would not be minded, the dental offices of this land would overflow with patients for months to come. It is the shrewdest form of dental business to avoid inflicting pain and no word in the Code prevents our exercising that skill and care in this respect which shall make our offices the Mecca of the fearful.

A certain dentist, then aged sixty years, located for practice in a large city where he was wholly unknown. It was his custom to assure a patient that no matter what he had to do he would not inflict pain. He kept his word. Within a short time he built up, entirely without advertising, a most desirable practice and retired ten years later, *having saved one hundred thousand dollars*. He attributes his success entirely to the fact that he never hurt a patient.

Fifth. He may do good work. This point needs no amplification. His fillings should, in the language of the common people, "stick." His upper plates should stay up and the lowers down. Taken in connection with an attractive personal appearance, a sympathetic manner and painless methods, a good quality of work is an excellent business builder.

Sixth. The dentist may make money. This is one of those duties toward himself concerning which the Code has nothing to say. It was no doubt the thought of its framers that the dentist would perceive the importance of this without legislation on the subject. If this was the thought of the fathers, it has been sadly twisted of late. There have arisen many well meaning but short-sighted men who seem to think that because it is not laid down in the Code that a dentist should make money, any activity tending to that end is unethical. They think that if the fathers had meant us to make money, they would have specified it in article number so-and-so; and since no article clearly sets forth such an aim in dentistry, it must have been the will of our professional ancestors that we should live and die poor.

There is nothing discernible in the Code which prevents a dentist being quite as keen a business man as the merchant. No word or line prevents his finding out what it costs him to conduct his practice and establishing fees which shall produce a desirable surplus annually. Nor is there any commandment that he shall not collect those fees as promptly as the merchant would in like case.

There is nothing in the Code which prevents the full and free discussion of the business side of practice in any society which elects to so discuss it. That many society men would strongly oppose such discussion is due to the fact that they have succeeded in forcing on the societies their own interpretations of the Code. Any society which chooses to indicate to such men that it desires to discuss the business side of practice, will be wholly within its rights under the Code. Incidentally, it will impart new vigor to its meetings, will greatly assist members needing this form of discussion and will draw to itself other dentists who now wait till the dry bones of theory are warmed here and there with a flush of life from practical subjects.

Letters in possession of the editor show that men everywhere are interested in the business subjects concerned with the practice of dentistry. Many of them would be glad to relegate to the scrap heap that interpretation of the Code which prevents such discussions. Let those who desire such discussions take hope. They have the right to them. It lacks only the courage on their part to bring them forward and insist on them.

Whatever may have been our previous conceptions, we find in the Code nothing expressed or implied which prevents our conducting our practices on profit-producing lines, so long as they are conducted on honor.

Seventh. The dentist is free, under the Code, to educate his patients to the appreciation of good dentistry. This means that he may tell his patients what good dentistry is and what it can do.

This does not mean that he shall unduly overrate himself. Assuredly it does not mean that he should belittle another. Mere worldly wisdom would indicate that the dentist refrain from self praise. It is an exhibition of weakness quickly perceived by others and never forgotten. It intimates that a man cannot safely leave it to others to praise him for his work, but that he fears it will not be done unless he seizes the opportunity to do it for himself.

The same form of wisdom shows the folly of belittling another or his work. Such a course reveals a mean soul. It déclares plainly that as he would take advantage of one who is not present to defend himself, so he would take advantage of the patient in those ways where the patient's knowledge is not sufficient to protect him. It labels the belittler as untrustworthy. This habit is a boomerang in the hands of him who practices it, and it often returns to wreak a damage on him who launched it.

That wisdom which looks only to results shows the advisability of refraining from criticism of the absent dentist or of making some excuse for his lack of success. It is far better to say "There are *now* methods which permit success in this work" than to say, "The chap who did that work did not know his business." One dentist somewhat given to such criticism was promptly and properly rebuked for it. A patient came to his chair with some bicuspid and molar fillings which were very black and which discolored the teeth. After examining the mouth he said, "That's very poor work in those silver fillings. If you'll let me replace them I'll put in some good ones." The patient said, "Do you know how long those fillings have been there?" "No," said the dentist. "Well sir, they were put in 22 years ago and the teeth are just as sound and comfortable as the day they were put in. You don't talk like the man I want to do my work." And with that he left the chair and office. Many a patient, not so out-spoken, has similar thoughts and leaves in like manner.

It makes a more favorable impression if we are big enough to stand on our merits without taking from our own pedestal a stone to shy at a fellow practitioner.

So when the Code restrains us from unfavorable comment on a fellow practitioner, it merely imposes that restraint which our own good sense will impose when we see our own welfare clearly.

Dentists have more useful information to impart to patients than is generally made use of. By giving it he elevates his profession and himself as its exponent. By such a course he endears himself to the patients who profit by his teaching, and secures their approval of him to their friends. They regard him as a dental authority. They quote him as having ability not common to other dentists. They seek his welfare and build his practice.

Eighth. The dentist is free to frequent public places of proper character and to bear himself with modest but attractive confidence. Many acquaintances begun in this way may be ripened into pleasant business relations. If he is asked, when among friends, to give information concerning the principles of his profession, the writer believes he should give it, keeping his personal connection with it out of the discussion. There is abroad in the land a desire for dental information, which no one seems willing to satisfy. It will be enough for the dentist at such time that he appears only as the giver of valuable information, the exponent of a useful gospel.

It is better form for the dentists to refer any inquiries which have to do with his services to his office during office hours. This imparts dignity to his position and brings to his office those who might not otherwise come. Under these conditions they are much more likely to be developed into patients.

Some of the activities which seem to be permitted under the Code may be tabulated as follows:

Exhibition of a pleasing personal appearance.
A clean and attractive and well kept office.
A courteous and winning manner.
Painless methods.
Good dentistry always.
Business methods as to fees and collections.
Education of patients to appreciate good services.
Proper appearances in public.

These things are the very elements of success. The de gree of their observance may be said to determine the success of each practitioner. Some dentists succeed by the exercise of one or two of them; as, for instance, by the exercise of good business ability and methods, with a limited amount of professional skill. Many a dentist has dragged out an unprofitable existence because he neglected some of these elements.

The writer has never known a case where all these elements were exercised, each in its proper place, that the dentist was not successful both professionally and financially. But where the dentist has felt that those things not expressly permitted in the Code were denied him on the pain of being unethical, he has often been a business failure.

These things permitted are the essence of tact in transforming the casual caller into the appreciative and constant patient. It matters little how many people come to an office if they go away without bestowing patronage.—*Dental Digest, Dec.* 1909.

